# The Substrate Versatility of Δ^1^-Pyrroline-5-carboxylate Reductase (ProC) from *Escherichia coli*

**DOI:** 10.3390/molecules31030501

**Published:** 2026-01-31

**Authors:** Eugenia Polverini, Alessandro Vecchi, Giulia Capra, Alessia Pastori, Alessio Peracchi

**Affiliations:** 1 Department of Mathematical, Physical and Computer Sciences, University of Parma, I-43124 Parma, Italy; eugenia.polverini@unipr.it; 2 Department of Chemistry, Life Sciences and Environmental Sustainability, University of Parma, I-43124 Parma, Italy; alessandro.vecchi2@studenti.unipr.it (A.V.); alessia.pastori@studenti.unipr.it (A.P.)

**Keywords:** substrate specificity, substrate docking, proline biosynthesis, imine reductase, multifunctionality

## Abstract

Δ^1^-Pyrroline-5-carboxylate reductase (EC 1.5.1.2; called ProC in most bacteria) is an enzyme of central metabolism that catalyzes the last step of the proline biosynthetic pathways, namely the NADPH-dependent reduction of pyrroline-5-carboxylate (P5C) to L-proline (L-Pro). The enzyme, however, is also active towards other substrates, and these reactions might have physiological relevance. Herein, the substrate versatility of ProC from *Escherichia coli* was explored as follows. We initially characterized the reverse reaction carried out by ProC, i.e., the formation of P5C from L-Pro. This reaction was easily measurable at pH 10, allowing the determination of the kinetic parameters. Under the same conditions, we then tested the ability of ProC to oxidize a number of L-Pro analogs, confirming that ProC reacts most effectively with analogs containing a simple five-membered ring such as L-thioproline (THP) and 3,4-dehydro-L-proline (DHP). Larger substrates such as L-pipecolate (PIP) reacted with lower efficiency, and the four-membered ring analog, L-azetidine-2-carboxylate (A2C) showed no detectable reactivity and behaved as a weak inhibitor of the ProC reaction. To interpret these results, we built a structural model of ProC and employed this model for a docking analysis of L-Pro and of its analogs. This approach highlighted the presence of a peculiar “three-point interaction”, in which the L-Pro carboxylate and amino groups form hydrogen bonds with conserved residues in the binding site, while the substrate ring stacks with the nicotinamide ring of NADP^+^. The L-Pro analogs tried to preserve as much as possible these critical interactions for a correct positioning and a favorable binding. The possibility of an inherent multifunctionality of ProC was further explored by examining the genomic context of the *proC* gene in a large number of bacterial species.

## 1. Introduction

Proline (L-Pro) is the only proteinogenic amino acid that contains a secondary α-amino group. In L-Pro, the nitrogen atom is locked into a pyrrolidine ring, which confers to the amino acid a peculiar rigidity and thereby affects the secondary structure of proteins in which L-Pro is included [1]. The biosynthesis of L-Pro is a highly conserved process that occurs in almost all species—from humans to plants and prokaryotes [2].

**Figure 1:**
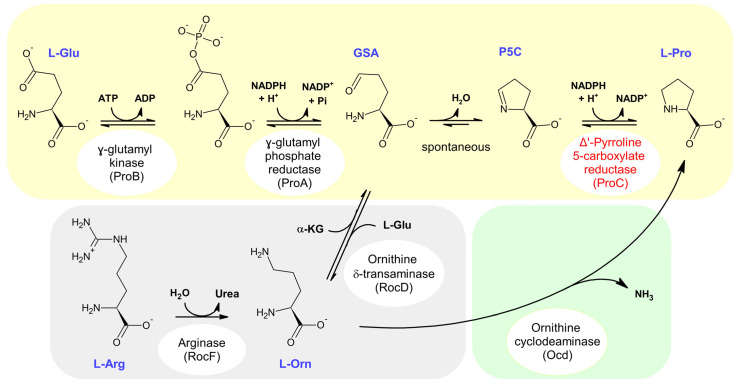
L-Pro biosynthesis pathways. In *E. coli* (reactions on yellow background) L-Pro is synthesized from L-glutamate. The consecutive reactions of γ-glutamyl kinase (ProB in *E. coli*) and γ-glutamate phosphate reductase (ProA) generate glutamate γ-semialdehyde (GSA). This compound cyclizes spontaneously to P5C, which is then reduced to L-Pro by P5C reductase (ProC). In different bacteria, such as. *B. subtilis*, L-Pro can be synthesized from L-arginine (gray background), which is converted into L-ornithine by arginase (RocF in *B. subtilis*). Ornithine can then yield P5C, through a reversible reaction catalyzed by ornithine-δ-aminotransferase (RocD). Completion of this pathway again requires the P5C reductase reaction (*B. subtilis* possesses at least two functional ProC homologs [5]). In a subset of bacteria, such as *Agrobacterium tumefaciens* (green background), L-Pro can be directly produced from L-ornithine through the activity of ornithine cyclodeaminase.

The metabolic routes for L-Pro biosynthesis are considered to originate from either L-glutamate or L-arginine [3,4] (Figure 1). In particular, the model organism *Escherichia coli* produces L-Pro from L-Glu in a pathway involving three enzymes (and one spontaneous reaction) (Figure 1, top). The last reaction in this pathway is the reduction of Δ^1^-pyrroline-5-carboxylate (P5C) to L-Pro, catalyzed by Δ^1^-pyrroline-5-carboxylate reductase (ProC). The synthesis of L-Pro from L-arginine, occurring for example in *Bacillus subtilis*, passes through the intermediate L-ornithine (Figure 1, bottom left), but again includes the formation of P5C and hence requires the activity of ProC. Only in a minority of organisms can L-ornithine be directly converted into L-Pro by the activity of ornithine cyclodeaminase (Figure 1, bottom right) [3].

Thus, in most cases, the routes of L-Pro biosynthesis merge at the level of P5C reductase (EC 1.5.1.2). This enzyme activity was first discovered about 70 years ago [6], and the ProC protein from *E. coli* was first characterized in the 1970s. ProC was reported to preferentially use NADPH as a coenzyme, in an apparently irreversible reaction [7].

Subsequently, ProC from *E. coli* was also shown to convert Δ^1^-piperideine-6-carboxylate (P6C) into L-pipecolate (PIP) [8]; this activity may be predominant in some ‘specialized’ homologs, such as APF3 from the fungus *Fusarium fujikuroi*, which is involved in the biosynthesis of the cyclic tetrapeptide apicidin F [9]. It was further shown that ProC can oxidize L-thioproline (THP; also called L-thiazolidine-4-carboxylate) [10]. THP is an L-Pro analog, containing a sulfur atom in the ring; it is derived from the nonenzymatic condensation between L-cysteine and formaldehyde [11], which in turn can originate from several intracellular processes [12,13].

These reported activities of *E. coli* ProC towards different substrates raise a number of questions. First, how broad is ProC’s acceptance of different substrates, and how efficiently does the enzyme process them? There has never been a systematic study on this point. Second, what are the structural bases for ProC substrate specificity? While there are a few experimental structures of bacterial ProC homologs [14,15], only one structure was solved with bound L-Pro [15]. And finally, does ProC’s activity towards different substrates have biological implications? Indeed, evolutionary biochemists distinguish between ‘promiscuous’ enzymes (those possessing side activities that are physiologically insignificant, e.g., because the alternative substrates are never available in the cell) and multifunctional enzymes (catalyzing multiple reactions that are biologically relevant) [16,17,18]. Identifying multifunctional enzymes seems important, for example, for building genome-scale metabolic models, which have been widely used to interpret and guide cell metabolic regulation [19].

The present study addresses the substrate scope of ProC, its structural basis, and its possible implications through a combination of biochemical approaches (steady-state kinetics on the purified *E. coli* enzyme) and bioinformatic analyses (structural prediction and molecular docking with the *E. coli* enzyme, as well as genome context analysis of bacterial *proC* genes).

## 2. Results

### 2.1. Reaction of ProC with L-Pro as a Function of pH and Coenzyme Type

The first objective of the present study was exploring the substrate versatility of ProC. Given the difficulty in comparing the reductase activity towards different oxidized substrates (several of which are not commercially available), we initially evaluated the possibility of studying the activity of ProC in its reverse reaction, the formation of P5C from L-Pro. In the first detailed study on this enzyme, Rossi et al. reported that the reverse reaction was not observable between pH 6.5 and 8, suggesting that the reduction of P5C (Figure 1) is essentially irreversible [7].

**Figure 2:**
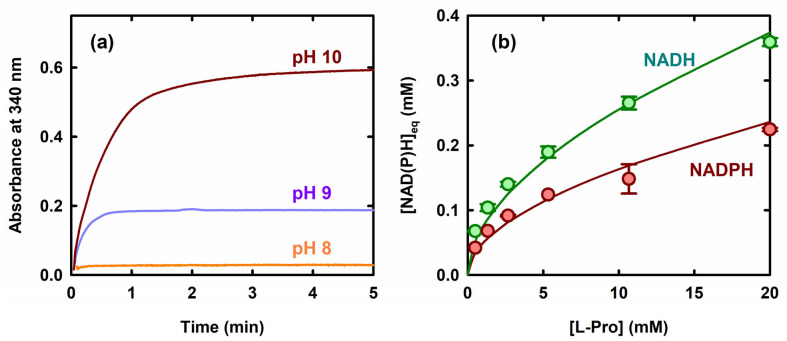
ProC-catalyzed L-Pro oxidation as a function of pH and of coenzyme type. (**a**) Formation of NADPH at three different pH values. Reactions were carried out in Bis-Tris propane buffer (50 mM, pH indicated in figure, room T) and were started by adding ProC (0.5 µM final concentration) to a solution containing 1.5 mM NADP^+^ and 10 mM L-Pro. (**b**) Amount of reduced coenzyme formed at equilibrium, as a function of the initial concentration of L-Pro. 50 mM Bis-tris propane buffer, pH 10, 27 °C. The solid curves through the data points are fittings to Equation (5) in Section 4.3, yielding for the reaction with NADP^+^ K′ = (1.5 ± 0.2) × 10^−3^ and for the reaction with NAD^+^ K′ = (3.8 ± 0.2) × 10^−3^.

Nonetheless, the reverse reaction (L-Pro oxidation with concomitant NADP^+^ reduction) is expected to become thermodynamically more favored as the pH increases, since a proton is released when NADP^+^ is reduced (Figure 1, upper right corner). Indeed, in studies on some ProC homologs, the oxidation of L-Pro could be measured at pH ~10 [20,21,22]. We hence initially tested this reaction in a pH range from 8 to 10, employing relatively high concentrations of L-Pro and NADP^+^ (10 mM and 1.5 mM, respectively). The results obtained (Figure 2a) revealed a significant accumulation of NADPH at pH 9, which further increased (about threefold) at pH 10. Overall, these observations show that ProC retains activity at pH 10, and at this pH value, the reverse reaction can be conveniently measured through a spectrophotometric assay that monitors NADPH formation.

We then studied in more detail the reaction at pH 10, using different concentrations of L-Pro and also changing the nicotinamide coenzyme (co-substrate). In these experiments, the L-Pro oxidation reaction appeared thermodynamically more favored when NAD^+^ was used. Indeed, the apparent equilibrium constant for the reaction (K′, calculated according to Equation (5) in Section 4.3) was ~2.5-fold higher when the oxidation of L-Pro was coupled to NAD^+^ reduction, as compared to NADP^+^ reduction (Figure 2b). This was consistent with literature data indicating that, from a thermodynamic point of view, NAD^+^ is reduced somewhat more easily than NADP^+^ [23,24].

### 2.2. Efficiency and Stereospecificity of the ProC-Catalyzed L-Pro Oxidation

While the functional unit of ProC proteins is a dimer, these enzymes have been reported to exist in different oligomeric states [15], also depending on the enzyme concentration [25]. When we tested the rate of the oxidation of L-Pro (10 mM) as a function of [ProC], the rate increased linearly with the enzyme concentration in the range of 0.07–0.7 µM, suggesting that in this interval, the quaternary assemblage of ProC was not changing (Figure S1). All subsequent kinetic experiments were conducted using ProC in this concentration range.

**Figure 3:**
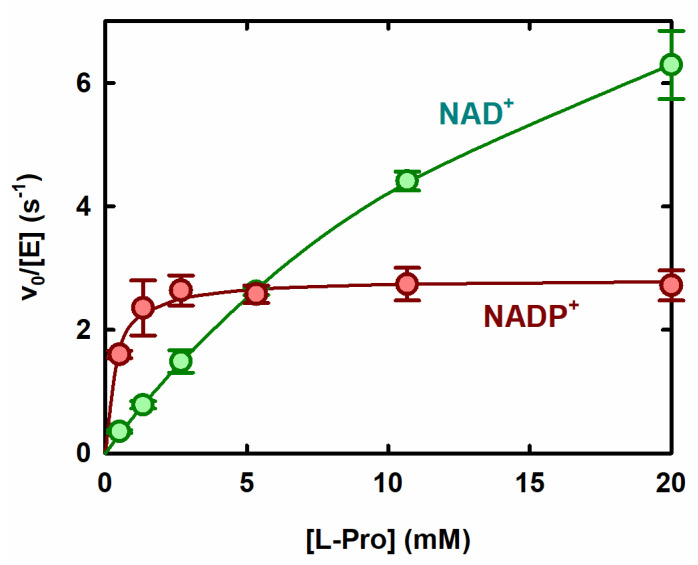
Rate of the ProC-catalyzed oxidation of L-Pro as a function of L-Pro concentration. 50 mM. Bis-tris propane buffer, pH 10, 1.5 mM NADP^+^ (red symbols) or NAD^+^ (green symbols), 27 °C. Solid lines represent nonlinear least-square fittings of the data to the Michaelis–Menten equation. The apparent kinetic parameters in the presence of NADP^+^ were k_cat_ = 2.8 s^−1^, K_M_ = 0.33 mM and k_cat_/K_M_ = 8500 M^−1^s^−1^; with NAD^+^, k_cat_ = 13 s^−1^, K_M_ = 20 mM, and k_cat_/K_M_ = 650 M^−1^s^−1^.

The ProC-catalyzed oxidation of L-Pro was then investigated by varying the substrate concentration and using either NAD^+^ or NADP^+^ as the coenzyme. With either electron acceptor, the reaction rate increased hyperbolically as a function of [L-Pro], as predicted from the Michaelis–Menten equation, with no evidence of cooperativity (Figure 3).

Notably, the efficiency of the reaction (expressed by the k_cat_/K_M_ parameter) was about tenfold greater in the presence of NADP^+^ than in the presence of NAD^+^. However, at high L-Pro concentrations (>5 mM), the reaction with NAD^+^ was faster than the reaction with NADP^+^, owing to a higher k_cat_ observed in the presence of the former coenzyme (Figure 3). This observation may have several explanations. For instance, if k_cat_ were partially limited by release of the reduced coenzyme, and if NADH were released considerably faster than NADPH, the turnover rate under saturating L-Pro conditions could be higher in the presence of NAD^+^.

To our knowledge, catalytic parameters for the oxidation of L-Pro had been previously measured only for ProC from *Mycobacterium tuberculosis* [22], which however was assayed under conditions significantly different from ours. The mycobacterial enzyme showed (at pH 10.3, 37 °C, using NADP^+^ as the coenzyme) a K_M_ comparable to that of the *E. coli* ProC (0.12 mM, vs. 0.33 mM in this study) but a 75-fold higher kcat (213 s^−1^ vs. 2.8 s^−1^) and a 200-fold higher k_cat_/K_M_ (1.77 × 10^6^ M^−1^s^−1^ vs. 8500 M^−1^s^−1^) [22].

In contrast to L-Pro, D-Pro (at concentrations up to 20 mM) was not appreciably oxidized by the *E. coli* ProC. This stereospecificity is similar to what was previously reported for a plant homolog [26] and consistent with the function of ProC in the synthesis of the proteinogenic L-Pro.

### 2.3. ProC Dehydrogenase Activity Towards L-Pro Analogs

**Figure 4:**
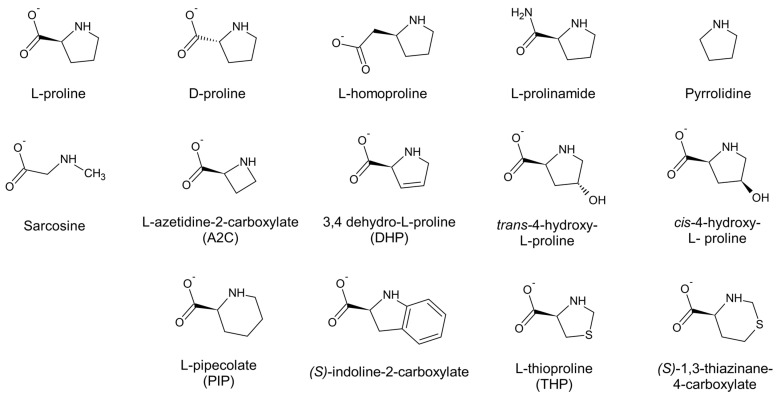
The substrates and potential substrates of ProC tested in this study. All the compounds contain a secondary amino group that can potentially be oxidized. For simplicity, the amino groups are shown in neutral form, even though at pH 10, they would generally exist as a mixture of protonated (charged) and unprotonated forms.

We next studied the ability of ProC to oxidize a number of (mostly natural) L-Pro analogs [27], adopting the same conditions used to study the oxidation of L-Pro (pH 10, 1.5 mM NADP^+^). The set of L-Pro analogs tested is shown in Figure 4.

Of these compounds, L-homoproline, pyrrolidine, sarcosine, L-azetidine-2-carboxylate (A2C), and indoline-2-carboxylate did not show any sign of reactivity. For the other compounds, we could measure oxidation kinetics (resulting in the formation of NADPH) and obtain apparent kinetic parameters, particularly k_cat_/K_M_, which can be used to compare the substrates’ reactivities [28]. In most cases, we could also establish an apparent equilibrium constant for the oxidation of these substances, much like what had been done for the oxidation L-Pro (see Figure 2b); this, however, was not possible for THP, thiazinane-4-carboxylate, and 3,4-dehydro-L-proline (DHP), as the reaction with these substrates was irreversible or quasi-irreversible. For the first two compounds, this might be attributed to the oxidation products undergoing further spontaneous transformations [11]; for DHP, the explanation was presumably the favorable aromatization of the pyrrolidine ring.

The results of the kinetic experiments with the reactive L-Pro analogs are summarized in Table 1. They suggest that the preferred ProC substrates contain, like L-Pro, a five-membered ring. The reaction with six-membered ring substrates (such as PIP) was evidently less efficient, and similarly, the two bulky 4-hydroxy-L-proline isomers, while both oxidized by ProC, reacted with a 150- to 300-fold lower k_cat_/K_M_ compared to L-Pro. The difference in reactivity was mostly attributable to a 100 to 200-fold higher K_M_ for the hydroxyprolines (Table 1), suggesting weaker binding of these compounds to the enzyme active site.

### 2.4. Assessing the Ability of the Unreactive L-Pro Analogs to Inhibit ProC

Analogs of L-Pro that were not substrates could be unreactive either because they did not bind to the ProC active site or because they did bind but could not be oxidized, or both. To assess whether such compounds do bind at the ProC active site, we tested their ability to act as inhibitors in the reaction of ProC with a good substrate (specifically, we used THP). D-Pro, L-homoproline, pyrrolidine, sarcosine, and L-indoline-2-carboxylate were not inhibitory at concentrations up to 20 mM, suggesting that they could not bind effectively at the active site of the enzyme. An exception was A2C, which gave a small but reproducible inhibition of the ProC reaction when used at 20 mM. Upon testing the compound at different concentrations (up to 78 mM), we estimated for A2C a Ki of ~50 mM, suggesting a weak affinity for the enzyme. By inference, the affinities of the other unreactive L-Pro analogs were presumably even weaker.

In addition to the compounds in Figure 4, two other L-Pro analogs were tested as possible inhibitors—namely, L-pyroglutamate (5-oxoproline) and ectoine. For chemical reasons, they could not be (and indeed were not) ProC substrates and also failed (at a concentration of 20 mM) to inhibit the enzyme.

### 2.5. Building a Structural Model for E. coli ProC

As no experimental structure of ProC from *E. coli* is currently available, building of a model was mandatory for the analysis of the enzyme active site and for interpreting its binding and reactivity properties.

The structure of P5C reductases consists of two domains, an N-terminal catalytic domain (pfam03807) and a C-terminal dimerization domain (pfam14748). The N-terminal domain consists of an α/β/α sandwich with a canonical NADP-binding Rossmann fold [15]. The C-terminal domain is rich in α-helices and is involved in a very interlaced domain swapping. The basic unit is, in fact, a swapped dimer that hosts two binding sites. Each site includes residues from both monomers and binds a NADP coenzyme.

The residues of *E. coli* ProC involved in substrate binding were deduced by similarity with the crystal structure of ProC from *Streptococcus pyogenes*, which contains the L-Pro ligand (see Section 4.5) [15]. These residues included Gly173, Ser174, and Met119 of one monomer, plus the NADP coenzyme, and residues Val229, Ser231, Thr235, and Thr236 of the other monomer.

A multiple sequence alignment of the *E. coli* ProC with structurally characterized homologs from the PDB database (see Section 4.5) revealed a high sequence conservation of the binding site (Figure S2). Of note, Nocek et al. [15], comparing the crystal structure of free ProC from *Neisseria meningitidis* with those of the *S. pyogenes* enzyme complexed with either NADP^+^ or L-Pro, did not observe any significant conformational differences in the active site, suggesting that binding of the substrates closely follow a lock-and-key mechanism. Based on this observation, any built model should maintain a high resolution at least at the active site, which is important for docking simulations.

A structural model of the *E. coli* ProC was then built by the Swiss-Model server [29] (see Section 4.5), which reports also the prediction of the oligomeric state of the protein. A slightly more reliable prediction for a homo-dimer quaternary structure (QSQE score of 0.68) than a homo-10-mer quaternary structure (QSQE score of 0.62) was obtained. For this reason, two models were eventually built (see Section 4.5). The first one was a homo-dimeric structure, based on the template of ProC from *Coxiella burnetii* (PDB code 3TRI [14], 36.7% sequence identity), crystallized with bound NADP^+^, that gave a high average model confidence [30] (QMEANDisCo score of 0.72).

The second one was a 10-meric model, built using as a template the structure of ProC from *S. pyogenes* (PDB code 2AMF [15] 34.38% sequence identity), again with a very good average model confidence (QMEANDisCo score of 0.69). This template is of particular interest because it crystallized with the ligand L-Pro, which was important for comparison with future docking results and, as stated above, for determining the ligand binding site.

The basic dimeric unit of the two models presented a very similar structure, even if the model based on the 2AMF template showed a more open conformation (Figure S3A). In both models, the C-terminal domain (residues 161 to 268) of each monomer strictly interlaces with the one in the other monomer and stabilizes the dimeric structure by means of strong hydrophobic interactions and a plethora of salt bridges, as reported for other structures of P5C reductases ([15] and references therein).

The structural alignment of the binding site residues of the two models was very good (RMSD = 0.79 on all atoms, Figure S3B). Therefore, we decided to test both models, docking the L-Pro ligand into the binding site and checking if the conformation and the key interactions reported in the literature [15] were maintained.

Beforehand, since the presence of the NADP coenzyme is essential for binding, we placed it in the right position inside the ProC structure via superimposition of each model with its template (or related template, see Section 4.5). To be consistent with the experimental conditions adopted in this study, we used the NADP^+^ form for the coenzyme, which is the same form available in each template. Subsequently, the NADP^+^ and its 4 Å surrounding region were energy minimized to optimize the geometry (Figure S4).

In subsequent docking simulations, the structures of enzymes and coenzymes were kept fixed.

### 2.6. Docking of L-Pro in the Two Different Models

In cases where experimentally solved structures are unavailable, homology modelling coupled with molecular docking has been widely adopted to predict ligand–target interactions (for a recent assessment of the approach, see [31]). The structure of L-Pro (extracted from the 2AMF template) was docked into the binding site of both models (see Section 4.6), and the resulting poses and binding energies were analyzed. The nitrogen of the L-Pro ligand was assumed to be protonated, in agreement with the predominant ionic form expected at the pH of our kinetic experiments (i.e., pH 10; the pK_a_ of the L-Pro amino group is 10.65 [32,33]).

**Figure 5:**
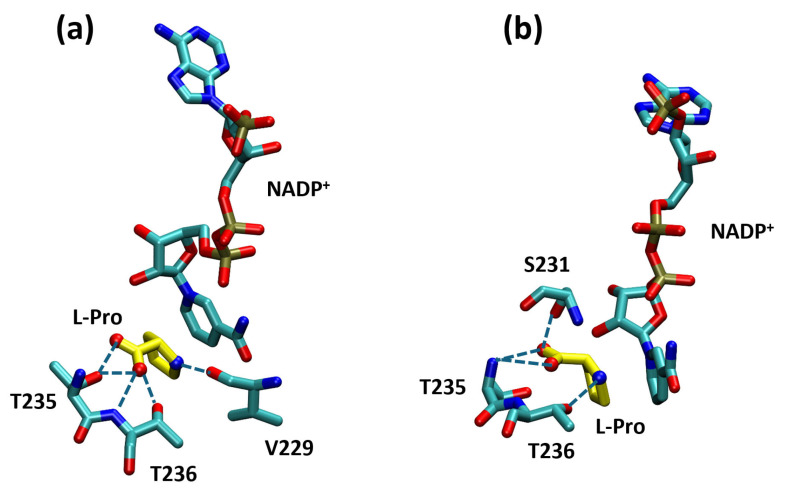
The predicted docking mode of L-Pro (yellow) in the ProC active site (cyan). The polar atoms are colored by type (red = oxygen, blue = nitrogen). (**a**) The key hydrogen bonding interactions reported by Nocek and co-workers [15] are indicated by dotted lines. (**b**) Shows the new interactions present in our docked model. In particular, stacking of the pyrrolidine ring of L-Pro with the nicotinamide ring of NADP^+^ is evident in both panels.

The calculated binding energies for the two complexes were very similar (−4.6 kcal/mol for the model built on the 3TRI template vs. −4.57 kcal/mol for the model built on the 2AMF template). In addition, for both models, the docking procedure reproduced the key interactions formed by L-Pro in the 2AMF structure [15]; namely, hydrogen bonds between the ligand carboxylate group and the OH group of Thr235 (a bond slightly longer for the model based on 2AMF), the OH group of Thr236 and the amide group of Thr236, as well as between the amino group of L-Pro and the carbonyl of Val229. However, docking to both models revealed three other H-bonds: two connecting the L-Pro carboxylate with the OH group of Ser231 and the amide group of Thr235, and a third bond between the L-Pro amino group and the OH group of Thr236. (Figure 5).

While Nocek and co-workers did not obtain a single structure of the enzyme co-crystallized with both NADP^+^ and L-Pro and hence only hypothesized an interaction of L-Pro with the coenzyme using structural superimposition [15], our docking simulations directly pointed to the stacking of the pyrrolidine ring of L-Pro on the nicotinamide ring of NADP^+^. More specifically, the two rings were slightly shifted to allow a cation–π interaction involving the protonated amino group of L-Pro. Apart from that, it is noteworthy that the contacts of L-Pro with the binding site did not include any salt bridges (involving, e.g., the carboxylate group) (Figure 5).

We must also stress that L-Pro reached its ‘correct’ position even though our docking did not include water molecules, which are present in the 2AMF structure and help with keeping the substrate in place [15].

Overall, as docking of the L-Pro substrate to the two models yielded analogous results, both models seemed legitimate starting points for docking simulations involving L-Pro analogs. However, the model based on the 3TRI template seemed to us somewhat superior on several grounds. First, it was complete at both termini, whereas the model based on the 2AMF template lacked the first two and the last three residues. Second, two loops in the model based on the 2AMF template had to be reconstructed (residues 85–88 and 27–30), with a loss of reliability. Third, ProC had a (slightly) higher sequence identity to the 3TRI template, and the corresponding model had a higher QMEANDisCo structural score (see above). Finally, and perhaps most importantly, the residues that in the 2AMF structure had been identified as important for formation of a decamer, helping to stabilize the dimer–dimer interface [15], are poorly conserved in the *E. coli* ProC. In particular, some residues that should constitute a hydrophobic patch are hindered in *E. coli* ProC by a basic arginine, while a histidine that should be stacking with the corresponding one in the neighboring dimer is substituted in the *E. coli* ProC by a glycine.

Therefore, based on these observations, we decided to continue our docking investigations employing the dimeric ProC model based on the 3TRI template.

### 2.7. Docking Analysis of Different L-Pro Analogs Binding to ProC

All the experimentally tested L-Pro analogs were docked on the binding site of the selected *E. coli* ProC model. As stated above (Section 2.5), the observation that the ProC binding site retains the same conformation in the absence and presence of the substrate L-Pro, even in different species, supported our approach of docking the L-Pro analogs on a rigid enzyme. For consistency with the experimental conditions, the prevalent ionization state for the amino group at pH 10 was considered for each compound (see Section 4.6). However, for substrates with a pKa in the interval of 10 ± 0.5, both forms were analyzed in our docking simulations. The calculated binding energies are reported in Table 2.

**Figure 6:**
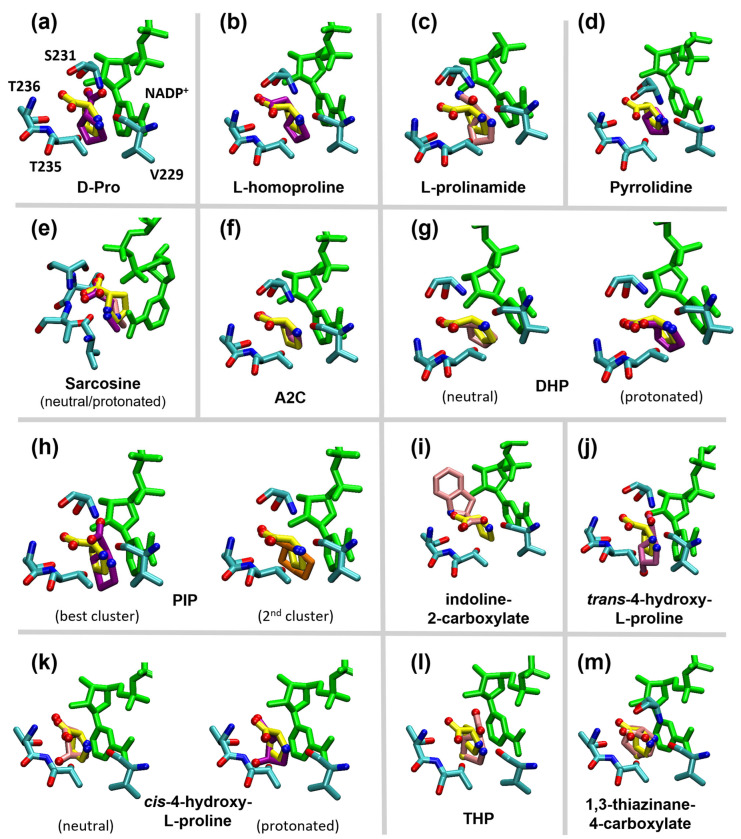
Docking modes of the L-Pro analogs in Figure 4, compared with L-Pro (yellow). Each panel (**a**–**m**) refers to a different analog, whose name is reported in the panel itself. The compounds docked with a neutral amino group are in pink, those with a charged amino group are in purple. For all analogs, the image shows the best-energy pose, except for PIP (panel (**h**)), where the pose representing the 2nd cluster is shown in orange. NADP^+^ (green) and the binding site residues (cyan) are labeled only in panel (**a**), for clarity. For all the molecules, except NADP^+^, the non-carbon atoms are colored by atom type (red = oxygen, blue = nitrogen).

For nearly all ligands, the best poses obtained from docking seemed to recapitulate the same interactions observed with L-Pro—namely, the multiple hydrogen bonds formed by the carboxylate and amino groups, as well as the stacking of the pyrrolidine ring on the nicotinamide ring of NADP^+^, in a sort of “three-point” interaction (Figure 6). However, the different chemical features of the compounds, as well as their different protonation states, led the molecules to assume slightly different positions in the active site, modifying the contributions of the individual groups to binding.

In particular, for D-Pro, the different chirality led to a predicted binding mode in which the carboxylate group formed no interactions, except for one with the Ser231 sidechain (Figure 6a). For indoline-2-carboxylate, owing to the presence of a large bicyclic ring, the best energy and most numerous cluster (almost 900 elements) showed a position on the edge of the L-Pro site (Figure 6i), while however preserving the carboxylate interactions with proteins Thr235 and Thr236.

For some other compounds, the presence of a neutral amino group reduced the ability to form H-bonds and prevented the occurrence of a cation–π interaction with the nicotinamide ring of NADP^+^. This resulted in a less favourable calculated binding energy, as compared to the case of a protonated amino group. Nevertheless, for most of these molecules, the “three-point” interaction mode was preserved, even better than in the protonated form.

The only exceptions were L-prolinamide, *trans*-4-hydroxy-L-proline, and THP (Figure 6c,j,l), which were predicted to tilt towards the NADP^+^ and found a more favorable position forming H-bonds with the 2′ oxygen of the nicotinamide nucleotide ribose and with the sidechain of Ser231. While for L-prolinamide, replacing the carboxylate with an amido group was apparently responsible for the tilt and for the new interactions, for *trans*-4-hydroxy-L-proline and THP, this could be due to the hydroxyl group present in the former compound and to the large sulfur atom in the latter. However, the new positioning results in a loss of the the carboxylate interactions with the Thr residues. Interestingly, the three molecules for which the two ionization states of the amino group are similarly probable at pH 10 (sarcosine, DHP, and *cis*-4-hydroxy L-proline; Figure 6e,g,k) showed almost identical poses in the neutral and protonated forms.

Among all reactive L-Pro analogs, the only ones for which the most populated cluster differed from the one with the best energy were thiazinane 4-carboxylate and *cis*-4-hydroxy-L-proline. For these compounds, the best pose reproduced the L-Pro conformation, but it was statistically unfavored. On the contrary, the most probable conformation showed a very distinct orientation or was even outside the binding site. This would suggest that correct binding at the active site is possible but not statistically favored, in tentative agreement with the modest reaction efficiency of these substrates. Docking on PIP, instead, retrieved two clusters close in energy and size, but with the ligand in a different orientation, the second pose resembling that of L-Pro (Figure 6h).

The stacking interaction with the nicotinamide moiety of NADP^+^ was preserved for all the reactive compounds. However, the different sizes and shapes of the rings appeared to slightly affect the distance and orientation with respect to the reactive C4 carbon of NADP^+^, potentially affecting electron transfer [34,35]. Considering the docked structures in Figure 6, the distance between the oxidizable carbon of the substrate (C5 in L-Pro) and the C4 carbon of NADP^+^ correlated poorly with k_cat_ (Figure S5a). The correlation was better when all reactive compounds were docked with a neutral nitrogen group (Figure S5b), which could suggest that the neutral form is the reactive one, similar to what has been described in other systems [36]. However this correlation should not be overinterpreted, because docking simulations cannot take into account several factors that can influence hydride tunneling for different compounds [34,35], such as protein dynamics or the role of water molecules in the active site, that cannot be modelled. Furthermore, k_cat_ may be limited in part by steps different from hydride transfer as well as by the occurrence of unproductive binding modes, as suggested by docking.

### 2.8. Exploring the Genomic Context of proC in Bacteria

The presence of P5C reductase in the vast majority of organisms attests to its key role in fundamental biological processes. While certainly L-Pro biosynthesis is the most obvious of such processes, the ability of ProC to transform other substrates (structurally related to L-Pro and P5C) may hint to possible further functions of the enzyme. To gain insights into this issue, we performed a survey of the gene context in which the *proC* gene occurs in bacterial genomes. This approach leverages the well-known phenomenon whereby bacterial genes involved in the same cellular function are commonly grouped in clusters or operons [37], a phenomenon driven by the advantages of co-regulation [38].

**Figure 7:**
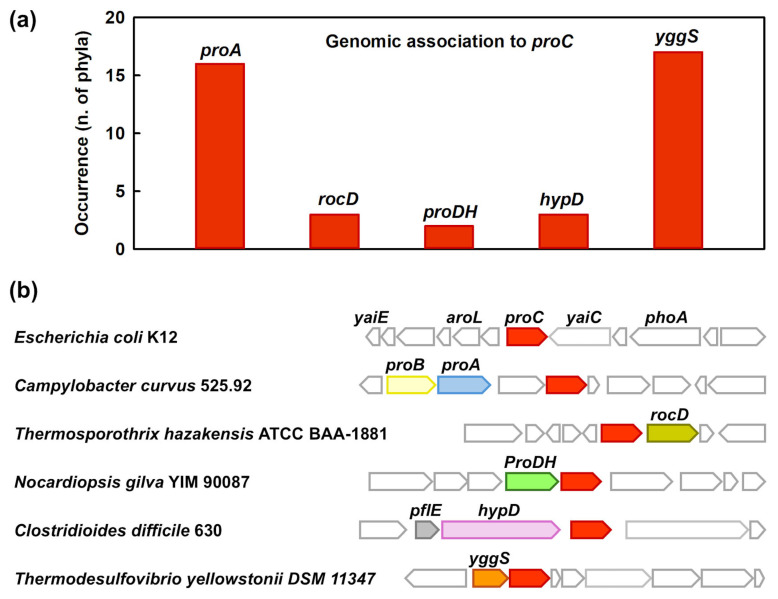
An overview of the genomic contexts in which *proC* gene homologs are found in bacteria. (**a**) Recurrence of associations involving *proC* in 42 different bacterial phyla. (**b**) Examples of *proC* neighborhoods from different bacterial genomes. In each example, the *proC* gene is shown in red. In *E. coli*, *proC* is not adjacent to other L-Pro related genes. In *Campylobacter* species, the association of *proC* with *proA* and *proB* is relatively frequent. The association with *rocD* is generally very rare, but it occurs for example in some Synergestota, such as *Thermosporothrix hazakensis*. The association with a gene encoding for L-Pro dehydrogenase (ProDH) is found in some Actinomycetota such as *Nocardiopsis gilva*. In *Clostridioides difficile*, whose genome includes two *proC* homologs, one of them is found next to *hypD*, a gene encoding *trans*-4-hydroxy-L-proline dehydratase. In many Nitrospirota (such as *Thermodesulfovibrio yellowstonii*), *proC* is associated with the gene of uncertain function *yggS*.

Gene clusters were examined in 42 bacterial phyla, through an approach described in the Section 4.7, with two main objectives. The first goal was to assess the frequence with which *proC* is genomically associated with other genes involved in the L-Pro biosynthesis pathways. The second goal was to highlight its possible recurrent association with clusters or enzyme-coding genes connected to other areas of metabolism. The most relevant results of this analysis are summarized in Figure 7a.

A reasonable expectation was that the *proC* gene would cluster preferentially with *proA* or *rocD*, encoding the enzymes that generate GSA (and hence P5C) in the two canonical L-Pro biosynthesis pathways (see Figure 1). However, such clusterings were not very common. We could find *proC* genomically associated with *proA* in just 16 bacterial phyla out of the 42 examined, whereas the *proC-rocD* association was much rarer (Figure 7a). Notably, in some phyla (such as *Thermodesulfobacteriota*), no association between *proC* and either *proA* or *rocD* was observed, even though several hundreds of genomes were examined. And, even in phyla where these associations were found, they were not particularly frequent. As an example, 23 genomes were examined from the phylum Bdellovibrionota, and the association *proC–proA* was found in only one of them (*proC–rocD* in none).

If *proC* is not clustering often with other canonical genes for L-Pro biosynthesis, what is it clustering with? In *E. coli* (where *proA* and *proB* are physically adjacent and evidently co-transcribed, even though the gene products do not form a stable complex [39]), *proC* is a ‘standalone’ gene (Figure 7b)—a situation that is far from uncommon. In other cases, a large variety of enzyme-coding genes (including an assortment of sugar kinases, methyltransferases, and aminoacyl-tRNA synthethases) were found in the vicinity of *proC*, in part depending on the specific phylogenetic branch. One notable instance, observed in several Actinomycetota and Pseudomonadota, was the association of *proC* with a gene encoding L-Pro dehydrogenase (ProDH; Figure 7b). This membrane-bound enzyme converts L-Pro to P5C in a FAD-dependent manner [40]. The coexpression of ProDH (which degrades L-Pro) with ProC (which synthesizes it) is counterintuitive but may suggest that some bacteria use L-Pro as a recyclable intermediate in the transport of electrons from NAD(P)H to an electron transport chain, much like in the ‘L-Pro cycle’ found in eukaryotes [4,41].

In some anaerobic bacteria such as *Clostridioides difficile*, *proC* homologs were found close to *hypD*, a gene encoding *trans*-4-hydroxy-L-proline dehydratase. This enzyme converts *trans*-4-hydroxy-L-proline (from food sources) into P5C [42]. In this case, ProC’s obvious function is to complete the recycling of the modified proline into plain L-Pro.

A particularly frequent association involved *yggS* (Figure 7a), a gene (conserved in a wide range of organisms) that was first characterized precisely as being co-transcribed with *proC* in *Pseudomonas putida* [43,44]. YggS and its homologs are involved in maintaining the homeostasis of vitamin B6 [45,46], and while they have no established enzyme function, a catalytic role seems likely. The *proC–YggS* association is found in at least 17 phyla and is almost the rule in some of them (e.g., Nitrospirota).

## 3. Discussion

### 3.1. Outlining the Substrate Preferences of E. coli ProC

ProC from *E. coli* can be considered the prototypical P5C reductase, having been studied since the 1970s. Nonetheless, the range of substrates that this enzyme can bind and transform has never been explored systematically. To address this issue, we expressed and purified to high levels a recombinant, His-tagged form of the enzyme. We then focused on the ‘reverse reaction’ catalyzed by ProC with L-Pro, identifying conditions where such a reaction could be conveniently studied. Under these conditions, we eventually investigated the substrate versatility of the enzyme, employing both natural and synthetic L-Pro analogs.

While we are aware that this study was conducted at an unusually high pH, which might influence substrate selectivity, the choice of studying the oxidation reaction was somewhat unavoidable, because (1) for some substrates, such as THP, the oxidation reaction is essentially irreversible, and (2) for other potential substrates, the oxidized form (e.g., the oxidized form of L-homoproline) was not commercially available. Furthermore, the predicted L-Pro binding site does not contain side chains that may be expected to change their ionization state between pH 8.0 and pH 10.0 (Figure 5).

The results of our experiments confirm that the preferred ProC substrates are five-membered rings with a carboxylate group adjacent to an amino carbon in the L-configuration. Of note, the carboxylate group of L-Pro could be converted into an uncharged (but nearly isosteric) amido group with only a 10-fold effect on reactivity (Table 1). In comparison, the substitution of the five-membered ring with a six-membered ring (PIP, thiazinane-4-carboxylate) or decoration with bulky substituents (the two 4-hydroxy-L-proline isomers), had significantly worse impacts on activity. A compound containing an even larger ring (indoline-2-carboxylate) was totally unreactive, and so were compounds containing a smaller, four-membered ring (A2C) or no ring (sarcosine). The unreactive analogs seemed unable to effectively compete with the substrate for binding at the active site.

### 3.2. Structural Basis of ProC Binding and Reactivity

Analysis of the ProC–L-Pro complex revealed a “three-point interaction” mode that appears crucial for binding and properly positioning the substrate: the L-Pro carboxylate interacts with the two conserved residues, Thr235 and Thr236; the substrate amino group interacts with the Thr236 sidechain and with Val229; finally, the pyrrolidine ring stacks against the nicotinamide ring of NADP^+^ (Figure 5).

When we examined the conformation of the L-Pro analogs docked in the ProC binding site, all the compounds apparently tried to preserve those critical interactions as much as possible. Nevertheless, the different chemical features of the compounds, as well as the protonation state of their amino groups, constrained the molecules in slightly different positions, affecting binding and substrate reactivity in different ways. As an example, L-homoproline arranged itself in order to preserve the carboxy and amino interactions, but, even if the pose was energetically favored, accommodation of the long tail caused a shift in the ring position that could make it less favorable for electron transfer (Figure 6b).

Overall, for most of the unreactive L-Pro analogs, the docking procedure indicated substantially weakened interactions or a distorted binding mode, or both. However, the correlation was far from perfect, suggesting that factors the docking procedure could not account for (such as the presence of active site water) may play a role in the binding and reaction of certain L-Pro analogs. In addition, we must consider that at pH 10, some amino acids sidechains could also change their ionization state—in particular, Cys and Tyr. Even if there are no such residues lining the L-Pro binding site (Figure 5), there are some residues of this kind that are significantly close to the site and to NADP^+^. Ionization of these residues at high pH might lead to local conformational changes in the protein, which could affect binding of certain substrates. On a possibly related note, Rossi et al. reported that ProC is inhibited by *p*-chloromercuribenzoate, a known blocker of Cys residues [7].

### 3.3. On the Biological Function of ProC

It is clear that, in bacterial genomes, the *proC* gene is most often not associated with other canonical genes involved in L-Pro biosynthesis, suggesting an independent and less restricted regulation. In part, this may mirror the fact that P5C can originate from additional reactions, besides those depicted in Figure 1—for example, HypD generates P5C from *trans*-4-hydroxy-L-proline [42], while ProDH obtains it from the oxidation of L-Pro [47]. Considering that other bacterial enzymes could produce P5C from yet different sources [48,49], and that the compound’s accumulation is reportedly toxic [50], ProC could be seen as playing a general detoxifying role towards P5C, not immediately connected to the need for L-Pro production.

A distinct (but not incompatible) possibility, however, is that ProC may be a multifunctional enzyme, involved in the transformation of a broader range of metabolic substrates, as also hinted by previous studies (e.g., [5]). With respect to this hypothesis, we note that some of the activities of ProC listed in Table 1 could in principle be biologically meaningful. As THP has been shown to be quite toxic, at least in mammalian cells [51], and since its oxidation by ProC has been shown to ultimately regenerate L-Cys (releasing formate) [11], this activity of ProC may be physiologically relevant. Indeed, it has been suggested that intracellular toxic levels of THP can be managed through oxidative metabolism thanks to the P5C reductase activity [10].

ProC also efficiently oxidizes DHP, a compound that has been occasionally found in nature, particularly in vegetables [52,53]. Notably, DHP’s predicted oxidation product should be pyrrole-2-carboxylate—a substance with a known but poorly understood microbial metabolism [54,55,56].

In vitro, the *E. coli* ProC also oxidized PIP appreciably (Table 1). In vivo, the enzyme has been shown to catalyze the opposite reaction, namely the synthesis of PIP through the reduction of P6C [8], which in turn may derive from the ε-transamination or oxidation of L-lysine. However, while the synthesis of PIP through P6C has been shown to be part of secondary metabolism in some fungi [9], its physiological relevance in bacteria remains uncertain [57,58].

Ultimately, establishing additional biological functions of ProC (and hence proving whether it is a multifunctional or just a promiscuous enzyme) will require in vivo approaches. In particular, metabolomics experiments on a Δ*proC E. coli* strain could highlight the possible accumulation of species such as THP, DHP, or P6C, in addition to P5C. The limits of such an approach should not be underestimated, however. For one, the intracellular concentrations of alternative substrates may be very low to begin with, and in the mutant, their accumulation may be curbed by metabolic rewiring or by functional redundancy [59]. For example, in *E. coli,* THP could be also consumed by PutA (proline utilization A), a FAD-dependent enzyme with L-Pro dehydrogenase and GSA dehydrogenase activities [60,61] that was shown to efficiently oxidize THP [62]. Furthermore, the findings in *E. coli* may not be readily extrapolated to other bacterial species, especially those possessing multiple *proC* homologs.

### 3.4. Final Remarks

We have shown that ProC from *E. coli* is able to bind and oxidize a series of compounds bearing structural and chemical similarities to L-Pro. The best alternative substrates, DHP, THP, and L-prolinamide, possess simple five-membered rings analogous to L-Pro. A docking study performed on a ProC model revealed that L-Pro establishes three crucial interactions with the binding site, namely H-bonds involving the carboxylate group, H bonds formed by the amino group, and stacking of the L-Pro ring. Differences in reactivity among the L-Pro analogs seemed partially related to the preservation of these interactions. For example, removal of the carboxylate group (in pyrrolidine) prevented reactivity, whereas the substitution of the carboxylate with an amido group (in L-prolinamide) had limited effects on both the H-bonding ability and on reactivity. Some of the observed reactions of ProC with alternative substrates could be physiologically relevant, although in vivo experiments seem required to clarify the issue. In any case, a survey of the genomic context of *proC* homologs in bacteria suggests that the function of ProC is not rigidly confined to the canonical L-Pro biosynthesis pathways.

## 4. Materials and Methods

### 4.1. Materials

*E. coli* ProC was obtained from an ASKA clone [63] as described [64] and purified using affinity chromatography on a His-Select^®^ cobalt affinity resin (Sigma-Aldrich-now Merck, Darmstadt, Germany) following the manufacturer’s instructions. Fractions with purity higher than 90% (as judged by SDS-PAGE) were pooled and dialyzed against storage buffer (50 mM potassium phosphate pH 7.5, 100 mM NaCl, 0.5 mM EDTA, 1 mM DTT). The purified, dialyzed enzyme was supplemented with 10% glycerol and stored at −80 °C.

Bis-Tris Propane, L-Pro, D-Pro, *trans*-4-hydroxy-L-proline (2S,4R), THP, and D,L 1,3-thiazinane-4-carboxylate were purchased from Sigma-Aldrich (now Merck, Darmstadt, Germany). Pyrrolidine was from Acros Organics (Geel, Belgium); *cis*-4-hydroxy-L-proline (2S,4S), L-β-homoproline, and (*S*)-indoline-2-carboxylate were from TCI (Tokyo, Japan); NADP^+^, A2C, and D,L-pipecolate were from Apollo Scientific (Manchester, UK); NADPH and PIP were from Alfa-Aesar (Karlsruhe, Germany); NAD^+^ was from Roche (Basel, Switzerland).

### 4.2. Kinetic Assays of ProC Activity

The kinetics of the ProC-catalyzed oxidation reactions were monitored spectrophotometrically by following the rise in absorption at 340 nm associated to the accumulation of NADPH (or NADH). The amount of NAD(P)H formed was quantitated from the observed changes in absorbance based on an extinction coefficient of 6220 M^−1^cm^−1^ for the reduced coenzymes.

Assays with L-Pro at different pH values (8–10) were conducted at 23 °C in 50 mM Bis-Tris propane buffer, using NADP^+^ and L-Pro at fixed concentrations (1.5 mM and 10 mM, respectively). In each assay, not only the pH of the buffer stock but also the pH of the L-Pro stock used had been adjusted beforehand at the desired final value. Reactions were started by adding ProC last, at a final concentration of 0.5 µM.

Steady-state kinetic parameters for L-Pro and its analogs were determined at pH 10.0 in Bis-Tris propane buffer (50 mM). The concentration of NAD(P)^+^ was kept fixed (1.5 mM), while the concentration of the second substrate varied from zero up to at least 20 mM. The concentration of ProC was typically 0.66 µM. All these measurements were performed in triplicate, and the data were plotted as the initial rate of NAD(P)H formation vs. the substrate concentration. The data were analyzed using nonlinear least-squares fitting to the Michaelis–Menten equation using Sigma Plot 14.0 (Systat Software Inc., San Jose, CA, USA).

### 4.3. Calculating the Apparent Equilibrium Constant for L-Pro Oxidation at pH 10.0

At pH 10.0, under the conditions described above, the amount of NAD(P)H formed varied as a function of the initial amount of L-Pro. This was consistent of course with the existence of an equilibrium for the L-Pro:NADP^+^ redox reaction, whose apparent equilibrium constant (K′) could be defined as follows:

(1) $$K^{\prime }=\frac{{{\left[P5C\right]}_{eq}\times \left[NADPH\right]}_{eq}}{{{\left[Pro\right]}_{eq}\times \left[NADP\right]}_{eq}}$$

where [P5C]_eq_, [Pro]_eq_, [NADPH]_eq_, and [NADP]_eq_ represent the equilibrium concentrations of the reagents and products. Based on Equation (1) and on the assumptions below:

(2) $${\left[NADP\right]}_{eq}={\left[NADP\right]}_{0}-{\left[NADPH\right]}_{eq}$$

(3) $${\left[Pro\right]}_{eq}={\left[Pro\right]}_{0}-{\left[P5C\right]}_{eq}$$

(4) $${\left[P5C\right]}_{eq}={\left[NADPH\right]}_{eq}$$

(where [NADP]_0_ and [Pro]_0_ represent the initial concentrations of NADP^+^ and L-Pro, respectively), the following equation was expected to describe the amount of NADPH formed at equilibrium as a function of the initial amounts of reagents:

(5) $${\left[NADPH\right]}_{eq}=\frac{-K^{\prime }\times \left({\left[Pro\right]}_{0}-{\left[NADP\right]}_{0}\right)+\sqrt{{\left(K^{\prime }\times \left({\left[Pro\right]}_{0}-{\left[NADP\right]}_{0}\right)\right)}^{2}+4\times K^{\prime }\times {\left[Pro\right]}_{0}\times {\left[NADP\right]}_{0}\left(1-K^{\prime }\right)}}{\left(2-2K^{\prime }\right)}$$

### 4.4. Estimating Inhibition Constants for Unreactive L-Pro Analogs

The L-Pro analogs that showed no apparent reactivity as substrates were tested as potential inhibitors under the same conditions. In these tests, THP was used as the substrate at a concentration below Km (1 mM), while the concentration of the postulated inhibitor was increased up to at least 20 mM. Each cuvette measurement was zeroed against blanks in which THP was omitted, and all assays were performed in triplicate. Initial velocities were calculated from the linear increase in absorbance over 1 min during the initial 2.5 min of the assay.

Decreases in activity were then fit to Equation (6), which assumes a purely competitive inhibition pattern:

(6) $$\frac{v}{v_{0}}=\frac{K_{I}(K_{M}+{\left[S\right]}_{0})}{K_{I}\left(K_{M}+{\left[S\right]}_{0}\right)+K_{M}[I]}$$

where v is the observed reaction rate, v_0_ is the rate in the absence of inhibitor, [I] is the concentration of inhibitor, [S]_0_ is the concentration of substrate (THP), K_M_ is the Michaelis–Menten constant for THP, and K_I_ is the inhibition constant for the unreactive analog.

### 4.5. ProC Structural Modeling

The Uniprot database [54] contained an AlphaFold model for the *E. coli* ProC (entry P0A9L8 [65]). However, this model represented a monomer, whereas the basic structural unit of ProC is a very interlaced swapped dimer in which each binding site comprises residues from both monomers. For this reason, and considering that the PDB databank [66] contains several structures of ProC homologs from different organisms, we resorted to building a model using the comparative modeling technique and the Swiss-Model server [29] in a user-supervised mode. As a note, the use of complexes built by the AlphaFold 3 server [67] was not permitted for docking purposes.

A BlastP similarity search [68] of the PBD database using the *E. coli* ProC sequence as a query retrieved 13 protein sequences. Multiple sequence alignment of those sequences plus the *E. coli* ProC was performed using Clustal omega [69] (Figure S2).

In Swiss-Model, the search for templates also returns a prediction of the oligomeric state of the protein based on an algorithm that combines interface conservation, structural clustering, and other template features to provide a quaternary structure quality estimate (QSQE) score [70]. The more reliable prediction was for a homo-dimer quaternary structure, with a QSQE score of 0.68. However, the homo-10-mer quaternary structure also had a good score (0.62).

Among the dimeric templates, considering both query coverage and sequence identity, the best was ProC from *C. burnetii* (PDB code 3TRI [14]), which contained bound NADP^+^. Therefore, a first model was built based on the 3TRI template (36.7% sequence identity), which gave a high average model confidence [30] (QMEANDisCo = 0.72).

However, a 10-meric model was also built, using as a template the structure of ProC from *Streptococcus pyogenes* (PDB code 2AMF [15]), crystallized with the ligand L-Pro. The sequence identity (34.38%) and the QMEANDisCo score (0.69) were slightly lower than for the previous model. The monomer structure predicted by AlphaFold was more similar to the model based on the 3TRI template (all atoms RMSD = 2.63) than to the model based on the 2AMF template (all atom RMSD = 2.93).

NADP^+^ was placed at the right position within each model structure by superimposing the model to its template. In the 2AMF template, NADP^+^ is absent, so we used the related structure 2AHR of the same protein, which contains the coenzyme [15] and is otherwise almost identical to 2AMF (all atoms RMSD 0.3 Å). Then, after fixing some steric clash through the Swiss-PdbViewer 4.1.0 software [71], NADP^+^ and a surrounding region of 4 Å were subjected to a light energy minimization using Chimera v. 1.17.3 [72] to optimize the structural geometry. Gasteiger partial atomic charges [73] were added to NADP^+^, which has a net charge of −3. The Amber ff14SB force field was used; minimization involved 100 steps of steepest descent plus by 10 steps of conjugate gradient.

### 4.6. Docking Simulations

After minimization, the ProC protein complexed with NADP^+^ was imported in the AutodockTool 1.5.7 (ADT) interface [74], preserving all hydrogens and charges. Nonpolar hydrogens were then merged in the united atom mode to prepare the receptor input file.

To match the experimental conditions, each ligand was built with either a charged or neutral amino group, depending on the prevalent ionization state at pH 10. If the pKa of the amino group was 10 ± 0.5, both forms of the compound were used for docking.

Except for L-Pro, whose coordinates were extracted from the X-ray crystal structure of the 2AMF template, the 3D structure of each substrate was built and downloaded via the Automated Topology Builder (ATB) database [75], and its optimized coordinates were used. All hydrogens and Gasteiger partial charges were then added using the Chimera 1.17.3 software, and the ligand was imported in ADT with no further modification except for the merging of non-polar hydrogens.

Docking simulations were performed with the Autodock 4.2 software package [74]. The grid box for the affinity maps was centered in the binding cavity and was 50 × 52 × 54 points with a spacing of 0.375 Å. All ligands had only 1 or 2 torsional degrees of freedom (DOF) that were set free to rotate. For each docking calculation, the Lamarckian Genetic Algorithm was used [76], performing 1000 runs with 27,000 generations, with an initial population of 150 individuals and with 2.5 × 10^6^ energy evaluations, to ensure docking convergence. A conformational cluster analysis was performed on the docked conformations, with a rmsd cluster threshold of 1 Å. Both the cluster with the best energy and the one with the highest number of conformations were analyzed. The structural analysis of the binding modes and interactions was performed with the softwares ADT 1.5.7, Chimera 1.17.3, VMD 1.9.3 [77], and Swiss-PdbViewer 4.1.0.

### 4.7. Genome Context Analysis

A preliminary survey of the genome context of the *proC* gene in different bacterial phyla was obtained by performing searches on the STRING website (https://string-db.org/) [78] using ProC from *E. coli* (NCBI: WP_001295331) as the query sequence. While STRING provides known and predicted protein–protein interactions based on different types of data, we set the gene neighborhood as the only interaction source.

Subsequently, as described in previous studies [79,80], the genomic context of *proC* homologs was explored in detail using tools available at the Integrated Microbial Genomes & Microbiomes (IMG/M) website (https://img.jgi.doe.gov/m/) [81]. Specifically, we visually inspected the contexts of *proC* homologs in over 1500 genomes annotated as ‘finished’ in IMG/M and belonging to 42 different phyla: Acidobacteriota, Actinomycetota, Aquificota, Armatimonadota, Atribacterota, Bacillota, Bacteroidota, Bdellovibrionota, Caldisericota, Calditrichota, Campylobacterota, Chlamydiota, Chlorobiota, Chloroflexota, Chrysiogenota, Coprothermobacterota, Cyanobacteriota, Deferribacterota, Deinococcota, Dictyoglomota, Elusimicrobiota, Fibrobacterota, Fusobacteriota, Gemmatimonadota, Ignavibacteriota, Kiritimatiellota, Lentisphaerota, Mycoplasmatota, Myxococcota, Nitrospinota, Nitrospirota, Planctomycetota, Pseudomonadota, Rhodothermota, Spirochaetota, Synergistota, Thermodesulfobacteriota, Thermodesulfobiota, Thermomicrobiota, Thermosulfidibacterota, Thermotogota, Verrucomicrobiota. In the case of phyla for which up to 200 ‘finished’ genomes were available, all the genomes were examined. In the case of phyla for which more than 200 genomes were available (e.g., Actinomycetota, Pseudomonadota) the examination was conducted on a sample of 200 to 400 genomes, trying to include the maximum diversity of species.

## Figures and Tables

**Table 1:** Apparent catalytic parameters of ProC towards the reactive L-Pro and analogs. Bis-Tris propane buffer pH 10.0, 27 °C. The reported parameters are averages (±SE) obtained from three separate experiments. Kinetic parameters in this table are deemed ‘apparent’ to denote the fact that they were collected at a fixed concentration of the NADP^+^ co-substrate (1.5 mM).

Substrate	k_cat_ (s^−1^)	K_M_ (mM)	k_cat_/K_M_ (M^−1^s^−1^)	K′
L-Proline	2.8 ± 0.1	0.33 ± 0.05	8500 ± 1400	(1.6 ± 0.1) × 10^−3^
L-Prolinamide	4.3 ± 0.6	5.2 ± 2	830 ± 220	(1.2 ± 0.1) × 10^−4^
3,4-dehydro-L-proline (DHP)	9.1 ± 0.9	0.54 ± 0.31	16,900 ± 7500	-
*trans*-4-hydroxy L-proline	1.1 ± 0.1	20 ± 3	55 ± 9	(1.0 ± 0.01) × 10^−4^
*cis*-4-hydroxy L-proline	1.5 ± 0.7	57 ± 35	27± 4	(1.1 ± 0.01) × 10^−4^
L-Pipecolate (PIP) *	0.6 ± 0.001	4.4 ± 0.6	140 ± 22	(1.0 ± 0.02) × 10^−4^
L-Thioproline (THP)	4.7 ± 0.4	2.3 ± 0.7	2100 ± 650	-
(*S*)-1,3-Thiazinane 4-carboxylate **	0.14 ± 0.01	2.5 ± 0.6	56 ± 14	-

* Essentially identical parameters were obtained using PIP in pure form or in a racemic mixture with D-pipecolate. This supported the notion that ProC is only reactive towards L-substrates and further suggested that D-pipecolate is not an inhibitor (or activator) of ProC. ** (*R*,*S*)-Thiazinane 4-carboxylate was used as the substrate. K_M_ and k_cat_/K_M_ were calculated assuming that only the (*S*) isomer (50% of the racemic mixture) was reacting.

**Table 2:** Calculated binding energies of L-Pro and its analogs, obtained through docking simulations. For each compound, the pKa of the amino group (either experimental or computed) served to estimate the major ionic form at pH 10, and such an ionic form was then employed for docking. For substrates with pK_a_ = 10 ± 0.5, binding energies for both ionic forms were calculated.

Ligand	pK_a_ of the Amino Group	Prevalent Ionization State of the Amino Group (pH 10)	Binding Energy (kcal/mol)
L-Proline	10.65	protonated	−4.6
D-proline	10.65	protonated	−4.2
L-homoproline	11.3	protonated	−5.0
L-prolinamide	8.9	neutral	−3.7
Pyrrolidine	11.31	protonated	−4.0
Sarcosine	10.01	protonated/neutral	−3.4/−2.7
L-azetidine-2-carboxylate (A2C)	10.7	protonated	−4.1
3,4-dehydro-L-proline (DHP)	9.57	protonated/neutral	−4.5/−3.5
*trans*-4-hydroxy-L-proline	9.4	neutral	−3.4
*cis*-4-hydroxy-L-proline	10.3	protonated/neutral	−4.6/−3.6
L-pipecolate (PIP) *	10.77	protonated	−4.7
Indoline-2-carboxylate	4.9	neutral	−3.7
L-thioproline (THP)	6.74	neutral	−3.2
(*S*)-1,3-Thiazinane 4-carboxylate	8.3	neutral	−3.6

* In the case of PIP, the values for the best energy cluster and for the 2nd populated cluster were identical within the first digit.

## Data Availability

The ProC structural models (with bound NADP^+^) used for the docking procedures are provided as Supplemental pdb files. The other data presented in this study are available upon request from the authors.

## Supplementary Materials

The following supporting information can be downloaded at: https://www.mdpi.com/article/10.3390/molecules31030501/s1, Supplementary figures: Figure S1: Dependence of the L-Pro oxidation rate as a function of ProC concentration; Figure S2: Multiple alignment of the sequences obtained by BLASTing the *E. coli* ProC sequence (in red) against the PDB database; Figure S3: Computed structures of ProC produced by the Swiss model server; Figure S4: Positioning and interactions of NADP at the ProC active site; Figure S5: relationship between k_cat_ and the calculated hydride donor–hydride acceptor distances in the docked enzyme–substrate complexes. Supplementary pdb files of the structural models used for docking: ProC_Model01-with-NADP_3tri-based.pdb and ProC_Model02-with-NADP_functional-dimer__2amf-based.pdb.

## Abbreviations

The following abbreviations are used in this manuscript:

P5C	Δ^1^-Pyrroline-5-carboxylate
GSA	L-Glutamate γ-semialdehyde
ProC	Δ^1^-Pyrroline-5-carboxylate reductase (from *E. coli*, if not otherwise specified)
THP	L-thioproline
DHP	3,4-dehydro-L-proline
A2C	L-azetidine-2-carboxylate
P6C	Δ^1^-piperideine-6-carboxylate
PIP	L-pipecolate
