# Supplementary material for: The Substrate Versatility of Δ1-Pyrroline-5-carboxylate Reductase (ProC) from Escherichia coli
Source: Molecules. 2026 Jan 31;31(3):501. doi: 10.3390/molecules31030501 (PMC12899673; doi:10.3390/molecules31030501)
Supplement: Supplementary file 1 [file molecules-31-00501-s001.zip › ProC_supplementary_figures.pdf]

molecules

Supplementary materials to:

The Substrate Versatility of Δ¹-Pyrroline-5-carboxylate Reductase (ProC) from *Escherichia coli*

Eugenia Polverini [1], Alessandro Vecchi [2], Giulia Capra [2], Alessia Pastori [2] and Alessio Peracchi [2,*]

[1] Department of Mathematical, Physical and Computer Sciences, University of Parma, I-43124 Parma, Italy; eugenia.polverini@unipr.it
[2] Department of Chemistry, Life Sciences and Environmental Sustainability, University of Parma, I-43124 Parma, Italy; alessandro.vecchi2@studenti.unipr.it (A.V.); alessia.pastori@studenti.unipr.it (A.P.)
* Correspondence: alessio.peracchi@unipr.it

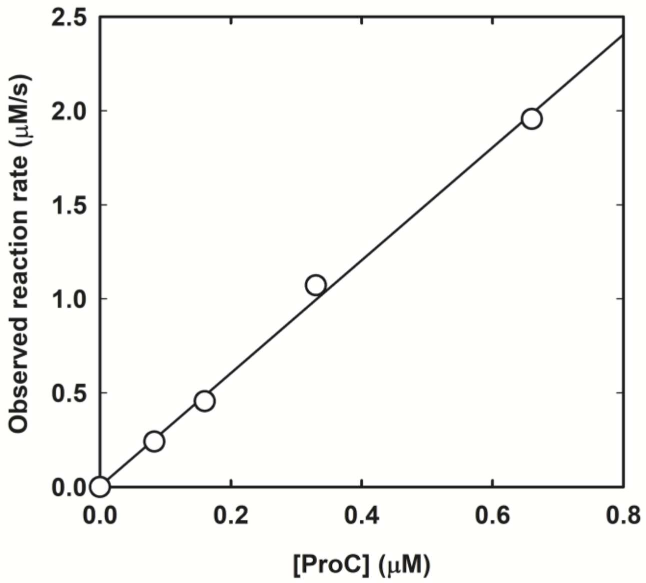

Figure S1. Linear dependence of the L-Pro oxidation rate as a function of ProC concentration. Conditions: L-Pro 20 mM, $NADP^+$ 1.5 mM, 50 mM Bis-tris propane buffer, pH 10, 25°C The enzyme concentration refers to the monomer.

```
tr|Q9K1N1|Q9K1N1_NEIMB    ----------MNVYFLGGGNMAAAVAGGLVKQGG---YRIYIANRGAE-KRERLEKELGV      46
tr|Q8IDC6|Q8IDC6_PLAF7    -------MENIKLGFMGLGQMGSALAHGIANANIIKKENLFYYGPSKK--------NTTL      45
tr|Q9A1S9|Q9A1S9_STRP1    ----------MKIGIIGVGKMASAIIKGLKQ----TPHELIISGSSLE-RSKEIAEQLAL      45
tr|Q83A21|Q83A21_COXBU    -------MNTSNITFIGGGNMARNIVVGLIANGY-DPNRICVTNRSLD-KLDFFKEKCGV      51
sp|P0A9L8|P5CR_ECOLI      --------MEKKIGFIGCGNMGKAILGGLIASGQVLPGQIWVYTPSPD-KVAALHDQFGI      51
tr|Q81C08|Q81C08_BACCR    --------MDKQIGFIGCGNMGMAMIGGMINKNIVSSNQIICSDLNTA-NLKNASEKYGL      51
tr|G7KRM5|G7KRM5_MEDTR    MEIIPIPADSYTLGFIGAGKMAESIAKGAVRSGVLSPSRIKTAIH-SNPARRTAFESIGI      59
sp|P32322|P5CR1_HUMAN     ----------MSVGFIGAGQLAFALAKGFTAAGVLAAHKIMASSPDMDLATVSALRKMGV      50
                                      : ::* *::.  :  *          .:                .  :

tr|Q9K1N1|Q9K1N1_NEIMB    ETSATLPE-LHSDDVLILAVKPQDMEAACKNIRT---NG-ALVLSVAAGLSVGTLSR---      98
tr|Q8IDC6|Q8IDC6_PLAF7    NYMSSNEELARHCDIIVCAVKPDIAGSVLNNIKPYLSSK--LLISICGGLNIGKLEE---      100
tr|Q9A1S9|Q9A1S9_STRP1    PYAMSHQDLIDQVDLVILGIKPQLFETVLKPLHF---K--QPIISMAAGISLQRLAT---      97
tr|Q83A21|Q83A21_COXBU    HTTQDNRQGALNADVVVLAVKPHQIKMVCEELKDILSETKILVISLAVGVTTPLIEK---      108
sp|P0A9L8|P5CR_ECOLI      NAAESAQEVAQIADIIFAAVKPGIMIKVLSEITSSLNKDSL-VVSIAAGVTLDQLAR---      107
tr|Q81C08|Q81C08_BACCR    TTTTDNNEVAKNADILILSIKPDLYASIINEIKEIIKNDAI-IVTIAAGKSIESTEN---      107
tr|G7KRM5|G7KRM5_MEDTR    TVLSSNDDVVRDSNVVVFSVKPQLLKDVVLKLKPLLTKDKL-LVSVAAGIKMK----DLQ      114
sp|P32322|P5CR1_HUMAN     KLTPHNKETVQHSDVLFLAVKPHIIPFILDEIGADIEDRHI-VVSCAAGVTISSIEKKLS      109
                                 :     :::. .:**         :     .    ::: . * .

tr|Q9K1N1|Q9K1N1_NEIMB    YLGGTRRIVRVMPNTPGKIGLGVSGMYAEAEVSETDRRIADRIMKSVGLTVWLDDEEKMH      158
tr|Q8IDC6|Q8IDC6_PLAF7    MVGSENKIVWVMPNTPCLVGEGSFIYCSNKNVNSTDKKYVNDIFNSCGI-IHEIKEKDMD      159
tr|Q9A1S9|Q9A1S9_STRP1    FVGQDLPLLRIMPNMNAQILQSSTALTGNALVSQELQARVRDLTDSFGS-TFDISEKDFD      156
tr|Q83A21|Q83A21_COXBU    WLGKASRIVRAMPNTPSSVRAGATGLFANETVDKDQKNLAESIMRAVGLVIWVSSEDQIE      168
sp|P0A9L8|P5CR_ECOLI      ALGHDRKIIRAMPNTPALVNAGMTSVTPNALVTPEDTADVLNIFRCFGE-AEVIAEPMIH      166
tr|Q81C08|Q81C08_BACCR    AFNKKVKVVRVMPNTPALVGEGMSALCPNEMVTEKDLEDVLNIFNSFGQ-TEIVSEKLMD      166
tr|G7KRM5|G7KRM5_MEDTR    EWAGHERFIRVMPNTAATVGEAASVMSLGGAATEEDANLISQLFGSIGK-IWKADDKYFD      173
sp|P32322|P5CR1_HUMAN     AFRPAPRVIRCMTNTPVVVREGATVYATGTHAQVEDGRLMEQLLSSVGF-CTEVEEDLID      168
                                 .:  * *    :  .         .          :  . *       :  :.

tr|Q9K1N1|Q9K1N1_NEIMB    GITGISGSGPAYVFYLLDALQNAAIRQGFDMAEARALSLATFKGAVALAEQTGEDFEKLQ      218
tr|Q8IDC6|Q8IDC6_PLAF7    IATAISGCGPAYVYLFIESLIDAGVKNGLSRELSKNLVLQTIKGSVEMVKKSDQPVQQLK      219
tr|Q9A1S9|Q9A1S9_STRP1    TFTALAGSSPAYIYLFIEALAKAGVKNGIPKAKALEIVTQTVLASASNLKTSSQSPHDFI      216
tr|Q83A21|Q83A21_COXBU    KIAALSGSGPAYIFLIMEALQEAAEQLGLTKETAELLTEQTVLGAARMALETEQSVVQLR      228
sp|P0A9L8|P5CR_ECOLI      PVVGVSGSSPAYVFMFIEAMADAAVLGGMPRAQAYKFAAQAVMGSAKMVLETGEHPGALK      226
tr|Q81C08|Q81C08_BACCR    VVTSVSGSSPAYVYMIIEAMADAAVLDGMPRNQAYKFAAQAVLGSAKMVLETGIHPGELK      226
tr|G7KRM5|G7KRM5_MEDTR    AITGLSGSGPAYIYLAIEALADGGVAAGLPRDLALSLASQTVLGAASMATQSGKHPGQLK      233
sp|P32322|P5CR1_HUMAN     AVTGLSGSGPAYAFTALDALADGGVKMGLPRRLAVRLGAQALLGAAKMLLHSEQHPGQLK      228
                            ..::*..*** :  :::: ...   *:    :  :   :. .:.     :      :

tr|Q9K1N1|Q9K1N1_NEIMB    KNVTSKGGTTHEAVEAFRRHRVAEAISEGVCACVRRSQEMERQYQ---------------      263
tr|Q8IDC6|Q8IDC6_PLAF7    DNIVSPGGITAVGLYSLEKNSFKYTVMNAVEAACEKSKAMGSK-----------------      262
tr|Q9A1S9|Q9A1S9_STRP1    DAICSPGGTTIAGLMELERLGLTATVSSAIDKTIDKAKSL--------------------      256
tr|Q83A21|Q83A21_COXBU    QFVTSPGGTTEQAIKVLESGNLRELFIKALTAAVNRAKELSKTVDQ--------------      274
sp|P0A9L8|P5CR_ECOLI      DMVCSPGGTTIEAVRVLEEKGFRAAVIEAMTKCMEKSEKLSKS-----------------      269
tr|Q81C08|Q81C08_BACCR    DMVCSPGGTTIEAVATLEEKGLRTAIISAMQRCTQKSVELSGQTKK--------------      272
tr|G7KRM5|G7KRM5_MEDTR    DDVTSPGGTTIAGVHELEKAGFRGILMNAVVAAAKRSQELS-------------------      274
sp|P32322|P5CR1_HUMAN     DNVSSPGGATIHALHVLESGGFRSLLINAVEASCIRTRELQSMADQEQVSPAAIKKTILD      288
                          . : * ** *  .:  :.   .   .  ..:     ::  :

tr|Q9K1N1|Q9K1N1_NEIMB    -------------------------------      263
tr|Q8IDC6|Q8IDC6_PLAF7    -------------------------------      262
tr|Q9A1S9|Q9A1S9_STRP1    -------------------------------      256
tr|Q83A21|Q83A21_COXBU    -------------------------------      274
sp|P0A9L8|P5CR_ECOLI      -------------------------------      269
tr|Q81C08|Q81C08_BACCR    -------------------------------      272
tr|G7KRM5|G7KRM5_MEDTR    -------------------------------      274
sp|P32322|P5CR1_HUMAN     KVKLDSPAGTALSPSGHTKLLPRSLAPAGKD      319
```

Figure S2. Multiple alignment of the sequences obtained by BLASTing the *E. coli* ProC sequence (in red) against the PDB database. The active site residues are highlighted in the ProC sequence of *E. coli* (yellow), *Coxiella burnetii* (green, corresponding to the 3TRI structure [1]) and *Streptococcus pyogenes* (cyan, corresponding to the 2AMF structure [2]).

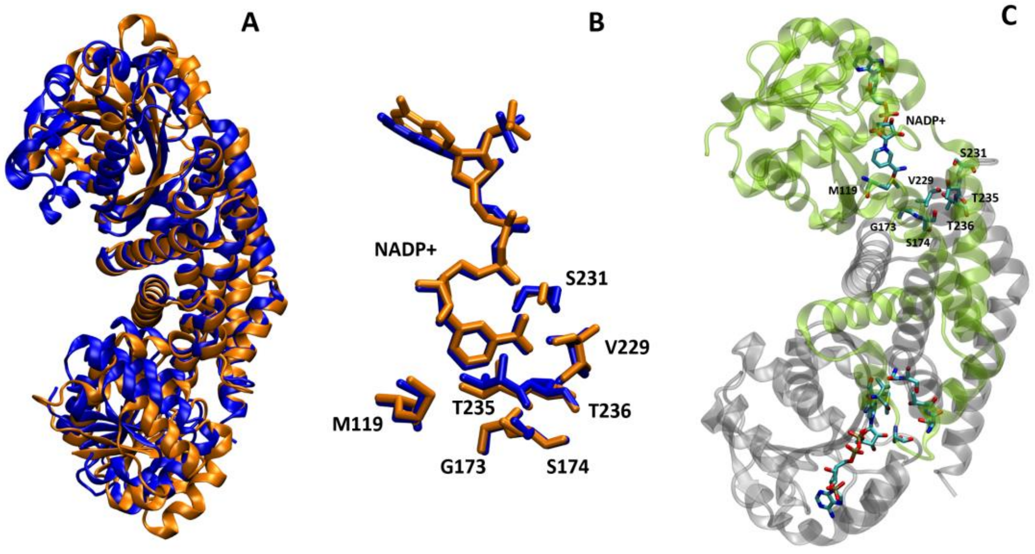

Figure S3. Computed structures of the *E. coli* ProC produced by the Swiss model server Panel A: structural alignment of the model based on the 3TRI template (blue) with the one based on the 2AMF template (orange). For the latter, only the dimeric functional unit is shown for comparison. Panel B: structural alignment of the active site alone (same color code as in panel A). Panel C: domain swapping of the dimeric unit of the 3TRI based model (in gray chain A and in green chain B), with the two «swapped» active sites in licorice, colored by atom type.

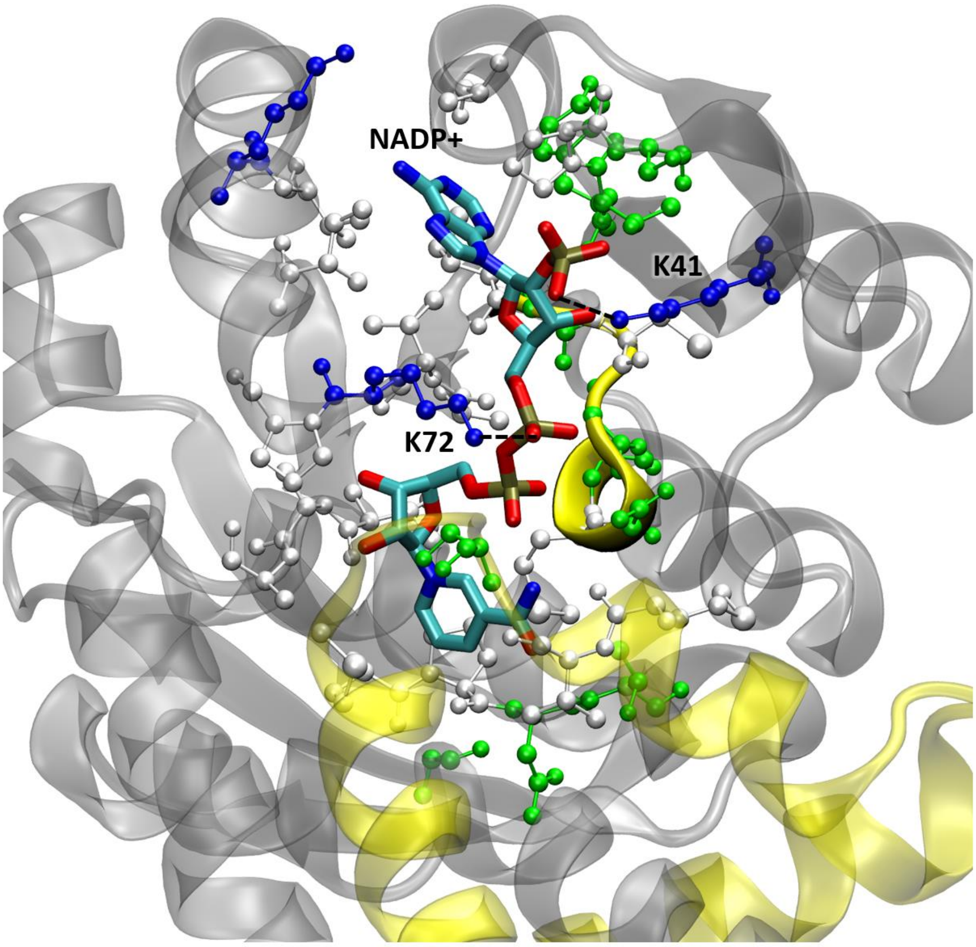

Figure S4. Positioning and interactions of NADP at the ProC active site. NADP+ is located inside a groove between the N-terminal domain and the jointed dimerization domains (in transparent gray and yellow the two swapped domains), with the nicotinamide ring forming a wall of the active center pocket. The typical dinucleotide-binding motif GXGXXA/G (residues 9-14, in opaque yellow), well conserved throughout the ProC enzyme family [2], is present. It forms a loop between strand 1 and helix 1 in the N-terminal domain and helps to maintain the correct position of the central part of NADP+. The coenzyme is held in place mainly by hydrophobic interactions (white ball-and-sticks residues) and by two salt bridges between the NADP+ phosphate groups and Lys41 and Lys72 in the ProC N-terminal domain. Interestingly, in the 3TRI template both these residues are conserved, whilst in 2AMF the first Lys is substituted with an Arg and the second one, although present, is far away from the NADP+ phosphate. Only a few H-bonds are formed by NADP+ with protein backbone atoms. It is worth noting that in the ProC crystal structures several water bridges mediate the interaction between protein and coenzyme, to stabilize the position of the latter.

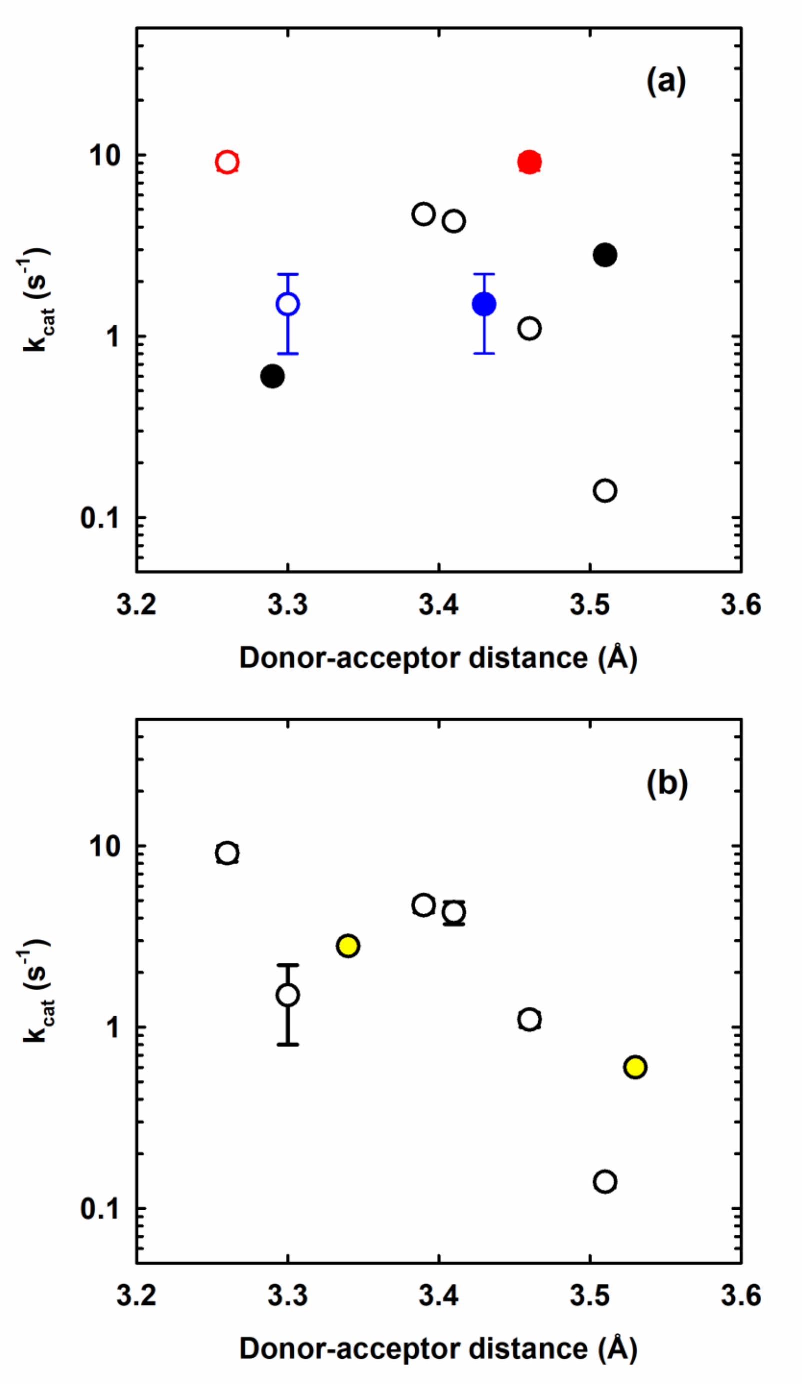

Figure S5. Representation of k_{cat} (values form Table 1 in the main text) as a function of the calculated distance between the hydride donor carbon on the substrate (e.g., C5 in the case of L-Pro) and the acceptor C4 atom on the nicotinamide ring. (a) The distances (measured from the best poses shown in Figures 5 and 6 of the main text) refer to substrates docked with a protonated (filled circles) or neutral (empty circles) amino group. For DHP (red) and cis-4-hydroxy-L-proline (blue) distances calculated for both the protonated and neutral forms are presented. (b) Correlation of k_{cat} with the distance of the best energy pose for compounds docked all with a neutral amino group. White circles correspond to the empty circles in panel a, whereas the yellow circles refer to L-Pro and PIP docked with an unprotonated amino group. With respect to the protonated form, the distance diminished for L-Pro and increased for PIP.

References

1. Franklin, M.C.; Cheung, J.; Rudolph, M.J.; Burshteyn, F.; Cassidy, M.; Gary, E.; Hillerich, B.; Yao, Z.K.; Carlier, P.R.; Totrov, M.; et al. Structural Genomics for Drug Design against the Pathogen *Coxiella Burnetii*. *Proteins: Structure, Function and Bioinformatics* 2015, *83*, 2124–2136.
2. Nocek, B.; Chang, C.; Li, H.; Lezondra, L.; Holzle, D.; Collart, F.; Joachimiak, A. Crystal Structures of Δ1-Pyrroline-5-Carboxylate Reductase from Human Pathogens *Neisseria Meningitides* and *Streptococcus Pyogenes*. *J Mol Biol* 2005, *354*, 91–106.
